# *CircRbms1* knockdown alleviates hypoxia-induced cardiomyocyte injury via regulating the *miR-742-3p*/*FOXO1* axis

**DOI:** 10.1186/s11658-022-00330-y

**Published:** 2022-03-26

**Authors:** Bo Liu, Kai Guo

**Affiliations:** Department of Cardiology, Xinhua Hospital, Shanghai Jiao Tong University School of Medicine, No.1665 Kongjiang Road, 20092 Shanghai, China

**Keywords:** Myocardial infarction, Hypoxia, *CircRbms1*, *MiR-742-3p*, *FOXO1*

## Abstract

**Background:**

Circular RNA (circRNA) has been shown to play an important role in a variety of cardiovascular diseases, including myocardial infarction (MI). However, the role of *circRbms1* in MI progression remains unclear.

**Methods:**

An MI mouse model was constructed in vivo, and cardiomyocytes were cultured under hypoxia condition to induce a cardiomyocyte injury model in vitro. The expression levels of *circRbms1*, microRNA (*miR)-742-3p*, and forkhead box O1 (*FOXO1*) were determined by quantitative real-time PCR. Cell viability, migration, invasion, and apoptosis were measured using Cell Counting Kit-8 assay, transwell assay, and flow cytometry. Meanwhile, western blot analysis was used to examine the protein levels of apoptosis markers and FOXO1. Additionally, dual-luciferase reporter assay, RNA pull-down assay, and RIP assay were employed to verify the interactions between *miR-742-3p* and *circRbms1* or *FOXO1*.

**Results:**

*CircRbms1* was upregulated in the heart tissues of MI mice and hypoxia-induced cardiomyocytes. Hypoxia induced cardiomyocyte injury by suppressing cell viability, migration, and invasion, and promoting apoptosis. Function experiments showed that *circRbms1* overexpression aggravated hypoxia-induced cardiomyocyte injury, while its silencing relieved cardiomyocyte injury induced by hypoxia. Furthermore, *circRbms1* sponged *miR-742-3p*. *MiR-742-3p* overexpression alleviated hypoxia-induced cardiomyocyte injury, and its inhibitor reversed the suppressive effect of *circRbms1* silencing on hypoxia-induced cardiomyocyte injury. Further experiments showed that *FOXO1* was a target of *miR-742-3p*, and its expression was positively regulated by* circRbms1*. The inhibitory effect of *miR-742-3p* on hypoxia-induced cardiomyocyte injury was reversed by FOXO1 overexpression.

**Conclusion:**

*CircRbms1 *regulated the *miR-742-3p*/*FOXO1* axis to mediate hypoxia-induced cardiomyocyte injury, suggesting that *circRbms1* might be an effective target for MI treatment.

**Supplementary Information:**

The online version contains supplementary material available at 10.1186/s11658-022-00330-y.

## Introduction

Myocardial infarction (MI) refers to the phenomenon of severe and persistent ischemia and necrosis of the myocardium caused by coronary artery occlusion [1, 2]. Clinically, MI is often accompanied by arrhythmia, heart failure, shock, and other complications, so it can seriously endanger the life of the patient [3, 4]. Many studies have shown that MI is mainly due to cardiomyocyte apoptosis leading to cardiomyocyte injury [5, 6]. Therefore, revealing the targets and potential molecular mechanisms that affect cardiomyocyte injury is of great significance for the development of new and effective MI treatment options.

Circular RNA (circRNA) with a covalently closed structure is a special type of noncoding RNA that is insensitive to nucleases and more stable than ordinary linear RNA [7, 8]. Emerging research confirms that circRNAs are becoming powerful regulators of human diseases [9, 10]. CircRNA has been found to be closely associated with malignant progression of cancer and can be used as a biomarker for cancer treatment [11, 12]. In addition, circRNA is also abnormally expressed in neurodegenerative diseases such as Alzheimer’s disease, and it has been confirmed to have an important function in disease development [13]. Importantly, circRNA also plays a key role in a variety of cardiovascular diseases, including MI [14]. For example, *circ_0060745* silencing was shown to relieve hypoxia-induced cardiomyocyte injury, alleviating MI [15].

In the GEO database (GSE133503), by analyzing the differentially expressed circRNA in the heart tissues of two controls and two MI mice, we found that *mmu_circ_0001022* (from *Rbms1* gene, also named *circRbms1*; the homologous circRNA in human is) was highly expressed in the heart tissues of MI mice. However, its role and function in MI had not been studied. Therefore, we chose circRbms1 as the object of this study to evaluate its role in MI by exploring its regulatory effect on hypoxia-induced cardiomyocyte injury. The proposed circRbms1/microRNA (*miR)-742-3p*/forkhead box O1 (*FOXO1*) axis improved the molecular mechanism of *circRbms1* regulating cardiomyocyte injury and provides a new potential target for the treatment of MI.

## Materials and methods

### MI mouse models

Twelve male C57BL/6 mice were purchased from Beijing HFK Bioscience Co., Ltd. (Beijing, China) and divided into two groups (*n* = 6 per group): MI group and sham group. All mice were housed with free access to food and water under standard conditions and subjected to a 12 h/12 h light/dark cycle. MI mouse models were constructed as previously described [16]. Briefly, C57BL/6 mice were anesthetized by intraperitoneal injection of 3% pentobarbital sodium (40 mg/kg; Sigma-Aldrich, St. Louis, MI, USA) and then the thoracic cavity was exposed. The left anterior descending coronary artery of the mice was ligated to construct an MI model. Sham group mice underwent the same surgery, but did not undergo ligation of the coronary arteries. After surgery, mice were kept separately and their health status was monitored daily. After 3 days, all mice were sacrificed to collect heart tissues. The infarct size of heart tissues was assessed by 2, 3, 5-trivinyltetrazolium chloride (TTC) staining (Solarbio, Beijing, China) and measured by Image J software. The animal study was approved by the institutional review board of Xinhua Hospital Affiliated to Shanghai Jiao Tong University School of Medicine (XHEC-JDYXY-2019-012) and was performed in compliance with the Basel Declaration. All animals received humane care according to the Guide for the Care and Use of Laboratory Animals.

### Cell culture

Mouse cardiomyocytes (H9c2) (ATCC, Manassas, VA, USA) were cultured in DMEM medium (Gibco, Carlsbad, CA, USA) containing 10% FBS (Gibco) and 1% penicillin–streptomycin (10,000 U/mL, Gibco) at 37 ℃ in a humidified incubator under hypoxia (2% O_2_) or normal (21% O_2_) conditions.

### Quantitative real-time PCR (qRT-PCR)

RNAsimple (Tiangen, Beijing, China) was used to isolate total RNA from heart tissues and H9c2 cells. Using the Transcriptor First Strand cDNA Synthesis Kit (Roche, Basel, Switzerland), the RNA was reverse transcribed into cDNA. The RT procedure was 37 ℃ for 15 min and 85 ℃ for 5 s. After that, SYBR Green PCR Kit (Takara, Dalian, China) was used for qRT-PCR in a PCR system. The amplification process was as follows: denaturation at 95 ℃ for 5 min, followed by 40 cycles at 95 ℃ for 15 s, annealing at 55 ℃ for 30 s, and extension at 60 ℃ for 60 s. *GAPDH* or *U6* was used as the internal control. Data were analyzed using the 2^−ΔΔCt^ method. The primer sequences were as follows: *circRbms1*, F 5′-CTGAGCCTGGACTCCATTCG-3′, R 5′-ACCAGGAGTTTCTGGTTATGGT-3′; *Rbms1*, F 5′-CTGAGCAAGACAAACCTCTACAT-3′, R 5′-GGCCTTATCCAAAATCGCCTT-3′; *miR-742-3p*, F 5′-GCCGAGGAAAGCCACCATGCTGG-3′, R 5′-CAGTGCGTGTCGTGGAGT-3′; *FOXO1*, F 5′-CCCAGGCCGGAGTTTAACC-3′, R 5′-GTTGCTCATAAAGTCGGTGCT-3′; *GAPDH*, F 5′-GGTGAAGGTCGGTGTGAACG-3′, R 5′-CTCGCTCCTGGAAGATGGTG-3′; *U6*, F 5′-CTCGCTTCGGCAGCACATATACT-3′, R 5′-ACGCTTCACGAATTTGCGTGTC-3′.

### Identification of circRNA circular characteristic

In RNase R assay, H9c2 cells were treated with RNAsimple to obtain RNA, and then the RNA was incubated with RNase R (Geneseed, Guangzhou, China) for 30 min. Nontreated RNA was used as mock. qRT-PCR was used to measure* circRbms1* and linear *Rbms*1 expression. In Actinomycin D (ActD) assay, H9c2 cells were incubated with ActD solution (R&D, Minneapolis, MN, USA) for 1 h. After further culturing for indicated times (0, 4, 8, and 12 h), the expression of *circRbms1* and linear *Rbms1* was determined by qRT-PCR.

### Cell transfection

For cell transfection, all oligonucleotides and vectors were synthesized from Ribobio (Guangzhou), including the circRbms1 small interference RNA (si-*circRbms1*) and its controls (si-NC), *miR-742-3p* mimic and inhibitor (*miR-742-3p* and anti-*miR-742-3p*) or their controls (miR-NC and anti-NC). The mimic and inhibitor of *miR-742-3p* were designed and synthesized by Sangon (Shanghai, China). The *circRbms1* overexpression vector and *FOXO1* overexpression vector was synthesized by subcloning a sequence of *circRbms1* and *FOXO1* into the pCD5-ciR vector and pcDNA3.1 vector, respectively. Lipofectamine 3000 (Invitrogen, Carlsbad, CA, USA) was used to transfect them into cells. The concentration of oligonucleotides was 50 nM, and the concentration of vectors was 4.0 µg. After transfection for 24 h, the cells were cultured under hypoxia for 24 h.

### Cell Counting Kit-8 (CCK8) assay

H9c2 cells were seeded into 96-well plates (5 × 10^3^ cells per well). After incubating for 48 h, H9c2 cells were incubated with CCK8 solution (Dojindo, Kumamoto, Japan) for 4 h. Then, cell viability was evaluated at 450 nm using a microplate reader (Bio-Rad, Hercules, CA, USA).

### Transwell assay

Transwell chambers (BD Biosciences, San Jose, CA, USA) precoated with Matrigel (BD Biosciences) were used for measuring cell invasion, and noncoated chambers were used for detecting cell migration. H9c2 cells were suspended with serum-free medium and then seeded on the upper chambers (2 × 10^5^ cells per well for cell migration and 4 × 10^5^ cells per well for cell invasion). The complete medium was added into lower chambers. Twenty-four hours later, the cells on the bottom of chambers were fixed with methanol (Beyotime, Shanghai, China) and stained with crystal violet (Beyotime). The migrated and invaded cell numbers were counted under a microscope (100×) (Leica, Wetzlar, Germany).

### Flow cytometry

Annexin V-FITC Apoptosis Detection Kit was obtained from Dojindo. According to the kit instructions, the H9c2 cell suspensions (5 × 10^5^ cells per mL) were suspended with binding buffer and incubated with Annexin V-FITC and propidium iodide. Cell apoptosis rate was analyzed by flow cytometry (Beckman Coulter, Miami, FL, USA). For detecting cell cycle process, the H9c2 cells were fixed with 70% alcohol and then treated with RNase A and propidium iodide. Cell cycle distribution was analyzed by flow cytometry.

### Western blot (WB) analysis

RIPA Lysis Buffer (Beyotime) was used to extract total protein, and BCA Protein Assay Kit (Beyotime) was used to quantify the protein. Afterwards, protein samples (30 µg) were separated by 10% SDS-PAGE gel and transferred to PVDF membranes (Beyotime). Next, the membranes were blocked with skimmed milk for 2 h. After incubating with primary antibodies against Bcl-2 (26Kda, 1:2,000, BA0412, Boster, Wuhan, China), Bax (20Kda, 1:1,500, BA0315-2, Boster), Cleaved-caspase 3 (17Kda, 1:1,000, AC033, Beyotime), FOXO1 (82Kda, 1:2,000, AF603, Beyotime), or GAPDH (36Kda, 1:2,000, A00227, Boster), the membrane was then incubated with HRP Conjugated AffiniPure Goat Anti-rabbit/mouse IgG (H + L) (1:10,000, BA1056, Boster). The protein signals were visualized using BeyoECL Star (Beyotime). EasySee Western Marker (25-90Kda, DM201-01, Transgen Biotech, Beijing, China) was used as a molecular weight standard.

### Dual-luciferase reporter assay

The sequences of *circRbms1* or *FOXO1* 3′UTR containing the predicted *miR-742-3p* binding sites were inserted into pGL3 vector (Promega, Madison, WI, USA) to build the wild-type (WT) vectors. The mutant-type (MUT) vectors were built in the same way. HEK 293 T cells (ATCC) were transfected with the *circRbms1*-WT/MUT or *FOXO1*-3′UTR-WT/MUT vectors and *miR-742-3p* mimic or miR-NC for 48 h. Dual-Luciferase Reporter Assay System (Promega) was used to detect the Firefly and Renilla luciferase activities to evaluate relative luciferase activity.

### RNA pull-down assay

H9c2 cells were transfected with biotin-labeled *miR-742-3p* probe (Bio-*miR-742-3p*) or negative control probe (Bio-miR-NC) (synthesized by Sangon). After 48 h, the cells were lysed and then the cell lysates were incubated with magnetic beads (Invitrogen) at 4 ℃ overnight. After purifying RNA, the enrichment of *circRbms1* was analyzed by qRT-PCR.

### RIP assay

According to the instructions of RNA Immunoprecipitation Kit (Sigma-Aldrich), H9c2 cells were lysed and then the cell lysates were incubated with magnetic beads conjugated with antibodies against IgG (anti-IgG) or Ago2 (anti-Ago2) overnight at 4 ℃. Then, qRT-PCR was used to determine the enrichment of *circRbms1* and *miR-742-3p*.

### Statistical analysis

Statistical analyses was conducted with GraphPad Prism 6.0 (GraphPad, La Jolla, CA, USA). The data are presented as mean ± standard deviation from three independent experiments. Significance of difference was determined using Student’s *t*-test or one-way analysis of variance followed by Tukey’s post-hoc test. *P* < 0.05 was considered statistically significant.

## Results

### *CircRbms1* was highly expressed in MI mice and hypoxia-induced H9c2 cells

**Fig. 1:**
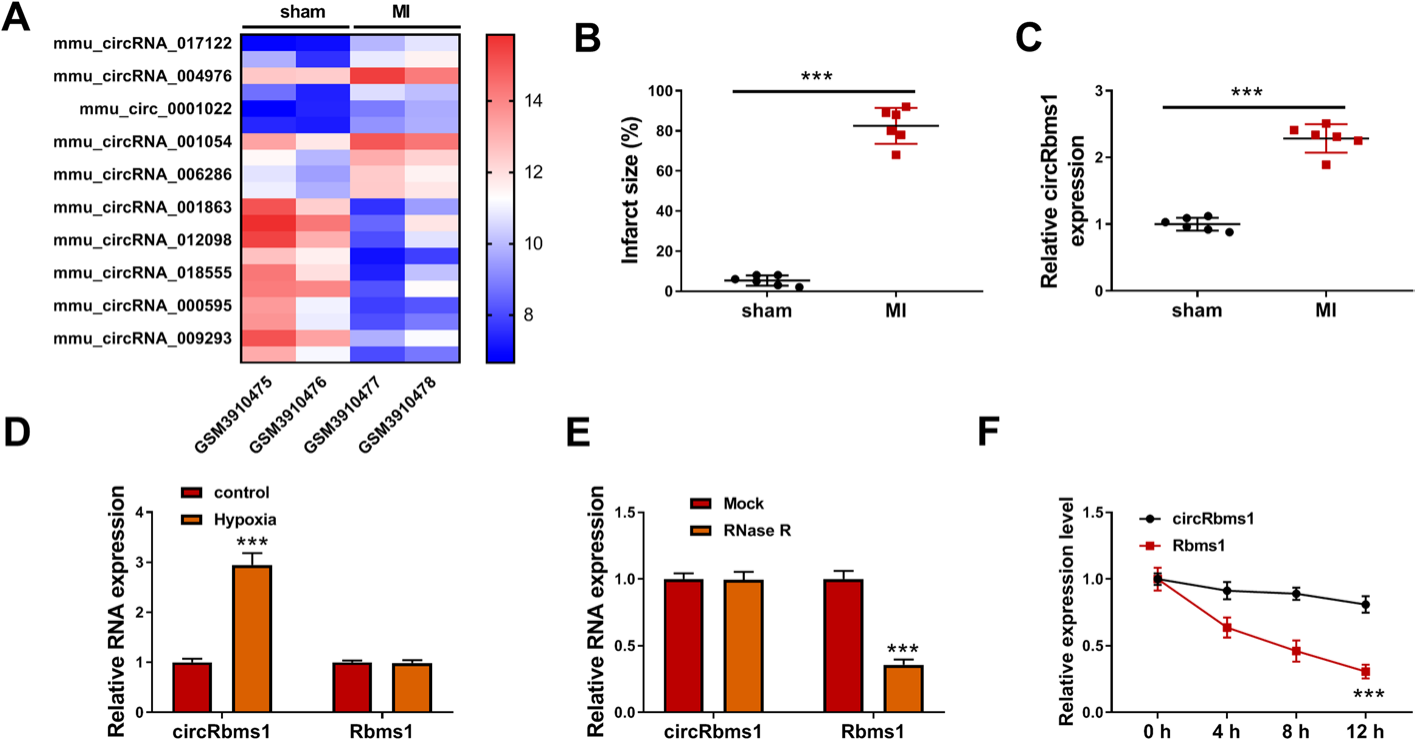
The expression of *circRbms1* in MI mice and hypoxia-induced H9c2 cells. **A** Heat map showing the differentially expressed circRNA in the heart tissues of two sham mice and two MI mice from the GEO database (GSE133503). **B** Infarct size in the sham mice and MI mice. **C** Expression of *circRbms1* in the heart tissues of sham mice (*n *= 6) and MI mice (*n* = 6), assessed by qRT-PCR. **D** Expression of *circRbms1* in hypoxia-treated and untreated H9c2 cells, assessed by qRT-PCR. **E**, **F** RNase R assay (**E**) and ActD assay (**F**) to confirm the circular characteristic of *circRbms1*. All experiments were repeated three times. ***P < 0.001

According to the cut-off criteria of values (*P* < 0.05 and |log2 fold change (log2FC)| > 1.0) in the GEO database (GSE133503), a total of 20 differentially expressed circRNAs were screened in the heart tissues of sham mice and MI mice, and *mmu_circ_0001022* (*circRbms1*) was an upregulated circRNA in MI mice (Fig. 1A). In constructed MI mice, we calculated the infarct size and found that the infarct size was significantly increased in the MI group (Fig. 1B). Using qRT-PCR, we discovered that *circRbms1* was indeed highly expressed in the heart tissues of MI mice compared with the sham group (Fig. 1C). In H9c2 cells induced by hypoxia, the expression of *circRbms1* was significantly increased, but the expression of linear *Rbms1* was not changed (Fig. 1D). To further confirm the circular characteristic of *circRbms1*, RNase R assay and ActD assay were performed, and the results showed that *circRbms1* could resist the digestion of RNase R and its expression was more stable than linear *Rbms1* (Fig. 1E, F). These data suggest that* circRbms1* is a stability circRNA and might play an important role in the progression of MI.

### Knockdown of *circRbms1* alleviated hypoxia-induced H9c2 cell injury

**Fig. 2:**
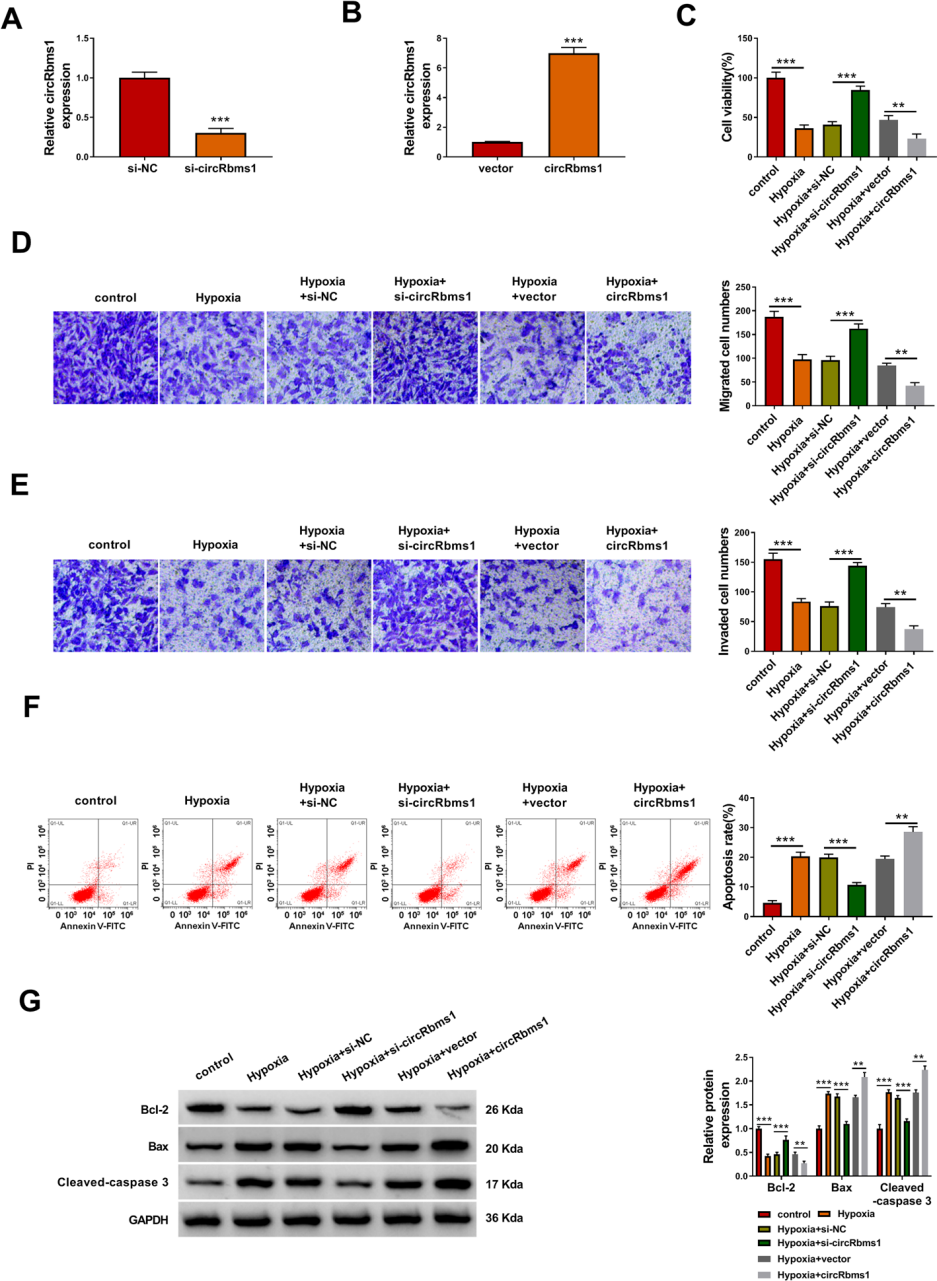
Knockdown of *circRbms1* alleviated hypoxia-induced H9c2 cell injury. **A**, **B** qRT-PCR was used to assess *circRbms1* expression to evaluate the transfection efficiency of si-circRbms1 (50 nM) or circRbms1 overexpression vector (4.0 µg) in H9c2 cells. **C**–**G** H9c2 cells were transfected with or without si-NC (50 nM), si-*circRbms1* (50 nM), vector (4.0 µg) or *circRbms1* (4.0 µg), and then treated with hypoxia. Untreated H9c2 cells were used as control. CCK8 assay (**C**), transwell assay (**D**, **E**) and flow cytometry (**F**) were used to determine cell viability, migrated and invaded cell numbers, and cell apoptosis rate, respectively. **G** WB analysis was performed to test the protein levels of Bcl-2, Bax, and Cleaved-caspase 3. All experiments were repeated three times. **P < 0.01, ***P < 0.001

To explore the role of *circRbms1* in MI progression, the siRNA and overexpression vector of *circRbms1* were constructed. After transfecting H9c2 cells with si-*circRbms1* and *circRbms1* overexpression vector, the expression of *circRbms1* was markedly decreased and increased, respectively (Fig. 2A, B). Then, H9c2 cells transfected with si-*circRbms1* and *circRbms1* overexpression vector were treated with hypoxia. By measuring cell viability, migrated cell numbers, and invaded cell numbers, we discovered that hypoxia could inhibit the viability, migration, and invasion of H9c2 cells (Fig. 2C–E). However, the inhibitory effect of hypoxia on H9c2 cell viability, migration, and invasion could be reversed by *circRbms1* silencing and aggravated by *circRbms1* overexpression (Fig. 2C–E). Besides, *circRbms1* knockdown also hindered the apoptosis rate of H9c2 cells promoted by hypoxia, while its overexpression enhanced hypoxia-induced H9c2 cell apoptosis (Fig. 2F). In addition, silenced *cicRbms1* also increased anti-apoptosis marker Bcl-2 protein expression and decreased apoptosis marker Bax and Cleaved-caspase 3 protein levels in hypoxia-induced H9c2 cells, while its overexpression had the opposite effect (Fig. 2G). We measured the cell cycle distribution in H9c2 cells in which *circRbms1* was silenced or overexpressed. Hypoxia increased the cell number in the G0/G1 phase and reduced the cell number in the S phase, indicating that hypoxia induced cell cycle arrest. Knockdown of *circRbms1* could promote the cell cycle in hypoxia-induced H9c2 cells, while its overexpression could aggravate cell cycle arrest (Additional file 1: Fig. S1A, B). All data reveal that *circRbms1* promoted hypoxia-induced H9c2 cell injury, suggesting that it might accelerate the progression of MI.

### *CircRbms1* directly sponged *miR-742-3p*

**Fig. 3:**
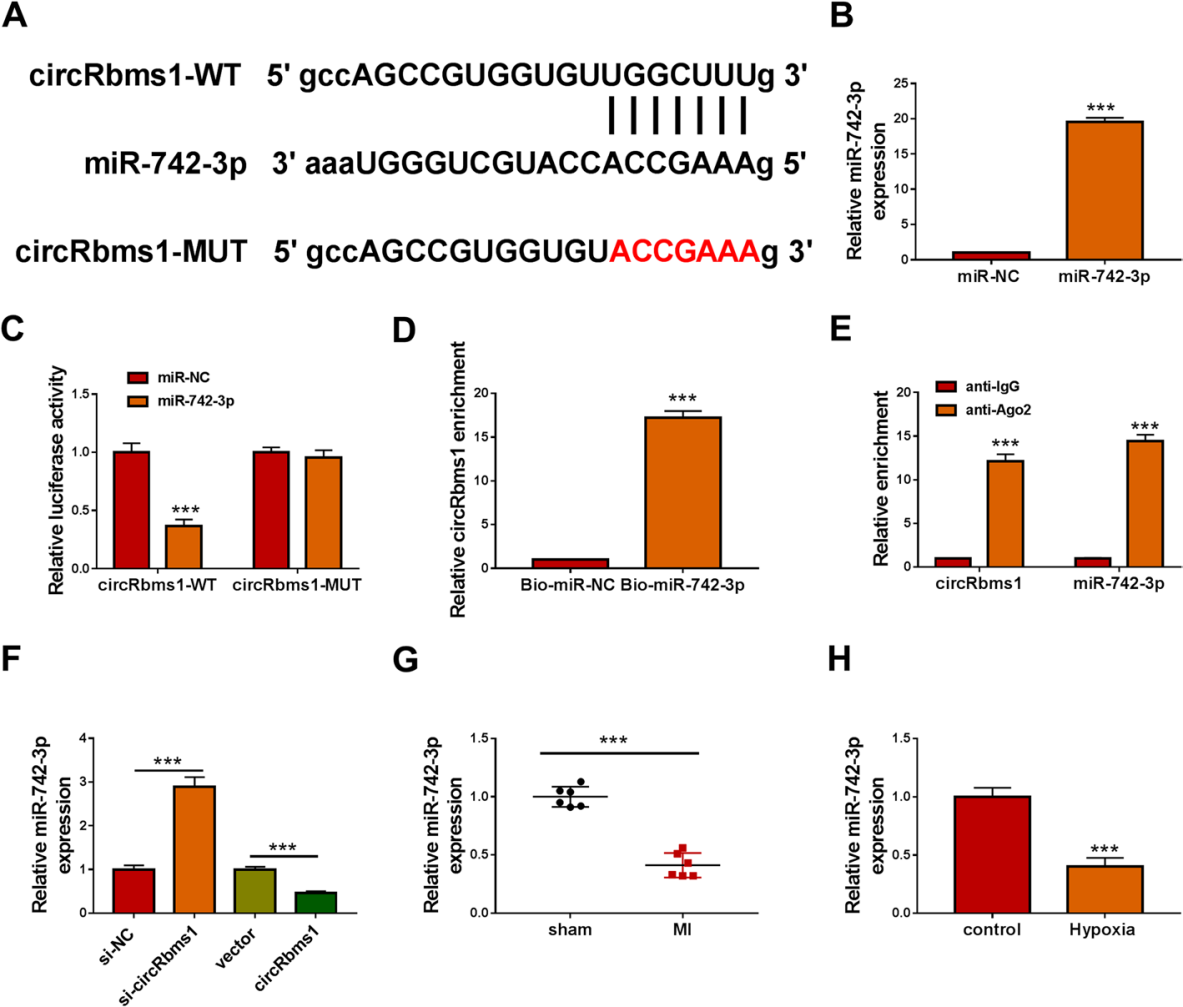
*CircRbms1* directly sponged *miR-742-3p*. **A** The fragments of *circRbms1*-WT and *circRbms1*-MUT. **B** The transfection efficiency of *miR-742-3p* mimic (50 nM) in H9c2 cells was confirmed by detecting *miR-742-3p* expression using qRT-PCR. Dual-luciferase reporter assay (**C**), RNA pull-down assay (**D**), and RIP assay (**E**) were used to verify the interactions between *circRbms1* and *miR-742-3p*. **F** In H9c2 cells transfected with si-NC (50 nM), si-*circRbms1* (50 nM), vector (4.0 µg), or *circRbms1* (4.0 µg), the expression of *miR-742-3p* was examined by qRT-PCR. **G** *MiR-742-3p* expression in the heart tissues of sham mice (*n* = 6) and MI mice (*n* = 6) was assessed using qRT-PCR. **H** The expression of *miR-742-3p* in hypoxia-treated and untreated H9c2 cells was measured by qRT-PCR. All experiments were repeated three times. ***P < 0.001

CircRNA has been shown to regulate cell biological functions as a competitive endogenous RNA (ceRNA) of microRNA (miRNA) [17, 18]. To investigate the mechanism of *circRbms1*, the Starbase tool (http://starbase.sysu.edu.cn/) was used to predict the targeted miRNA for *circRbms1*. Analysis revealed the presence of *miR-742-3p* complementary binding sites on* circRbms1* (Fig. 3A). Subsequently, *miR-742-3p* mimic was built to perform function experiments. The transfection efficiency of *miR-742-3p* mimic was confirmed by detecting its expression after transfection (Fig. 3B). Then, the results of dual-luciferase reporter assay showed that the luciferase activity of *circRbms1*-WT vector could be inhibited by *miR-742-3p* overexpression, while that of the *circRbms1*-MUT vector had not changed (Fig. 3C). RNA pull-down assay indicated that the enrichment of *circRbms1* was markedly increased in the Bio-*miR-742-3p* probe compared with the Bio-miR-NC probe (Fig. 3D). Also, RIP assay results suggested that the expression of *circRbms1* and* miR-742-3p* could be enriched in anti-Ago2 (Fig. 3E). These data confirmed that there was an interaction between *circRbms1* and *miR-742-3p*. Further experiments revealed that* miR-742-3p* expression was promoted by *circRbms1* knockdown and repressed by *circRbms1* overexpression (Fig. 3F). In the heart tissues of MI mice and hypoxia-induced H9c2 cells, we found that *miR-742-3p* expression was significantly lower compared with the corresponding controls (Fig. 3G, H).

### *MiR-742-3p* could relieve hypoxia-induced H9c2 cell injury

**Fig. 4:**
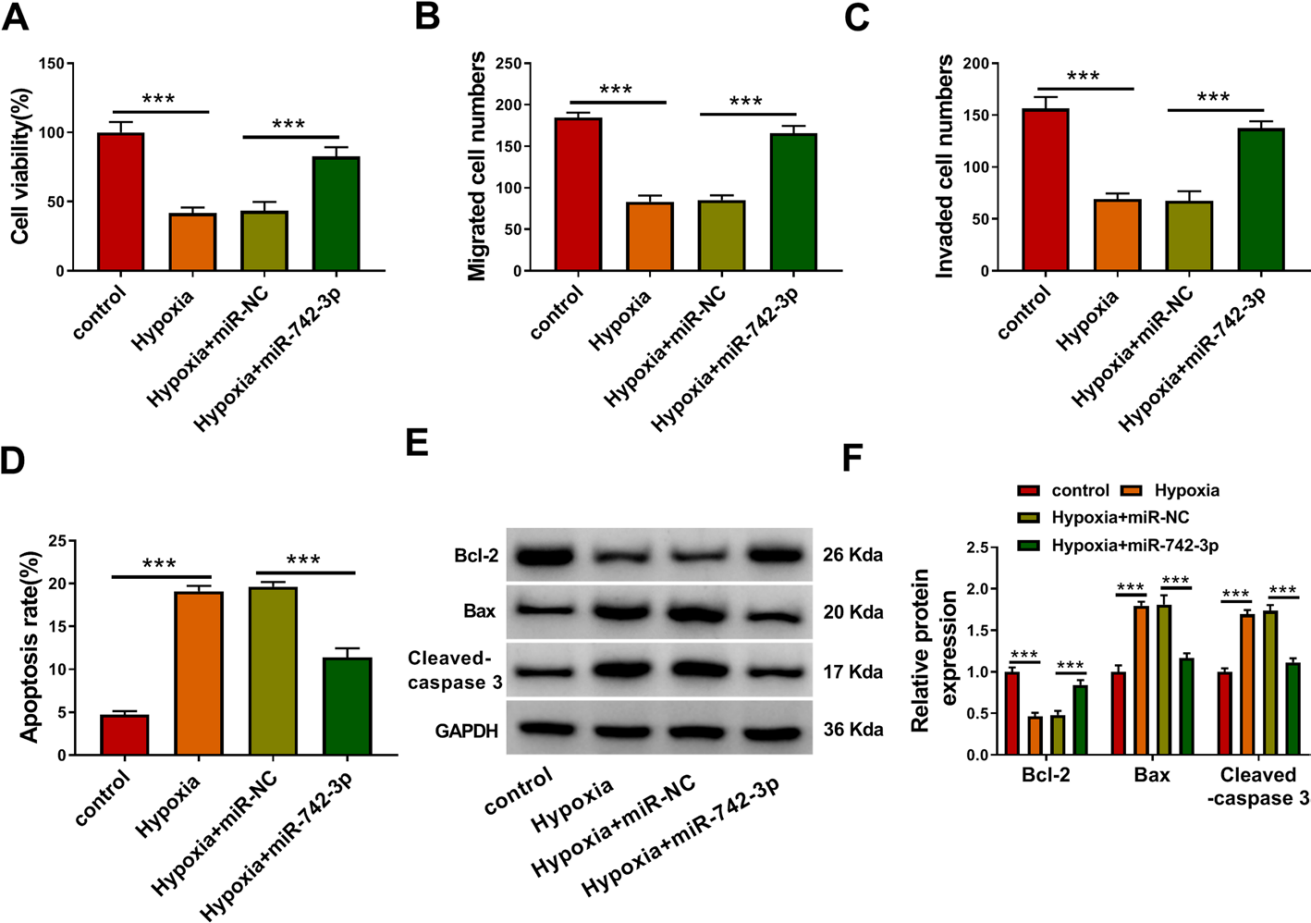
*MiR-742-3p* relieved hypoxia-induced H9c2 cell injury. H9c2 cells were transfected with or without miR-NC (50 nM) or *miR-742-3p* mimic (50 nM), and then treated with hypoxia. Untreated H9c2 cells were used as control. Cell viability, migrated and invaded cell numbers, and cell apoptosis rate were measured using CCK8 assay (**A**), transwell assay (**B**, **C**) and flow cytometry (**D**). **E**, **F** The protein levels of Bcl-2, Bax and Cleaved-caspase 3 were determined using WB analysis. All experiments were repeated three times. ***P < 0.001

To confirm the function of *miR-742-3p* in MI, we evaluated its regulation on hypoxia-induced H9c2 cell injury. The results showed that *miR-742-3p *overexpression reversed the suppressive effect of hypoxia on the viability, migration, and invasion of H9c2 cells (Fig. 4A–C). Furthermore, *miR-742-3p* also inhibited the apoptosis rate of H9c2 cells induced by hypoxia (Fig. 4D). The increased Bcl-2 protein level and the decreased Bax and Cleaved-caspase 3 protein levels in the presence of *miR-742-3p *in hypoxia-induced H9c2 cells also indicated that *miR-742-3p* could recover the apoptosis-promoting effect of hypoxia on H9c2 cells (Fig. 4E, F). These results reveal that *miR-742-3p* could protect cardiomyocytes from hypoxia-induced injury.

### *MiR-742-3p* inhibitor reversed the inhibitory effect of *circRbms1* silencing on hypoxia-induced H9c2 cell injury

**Fig. 5:**
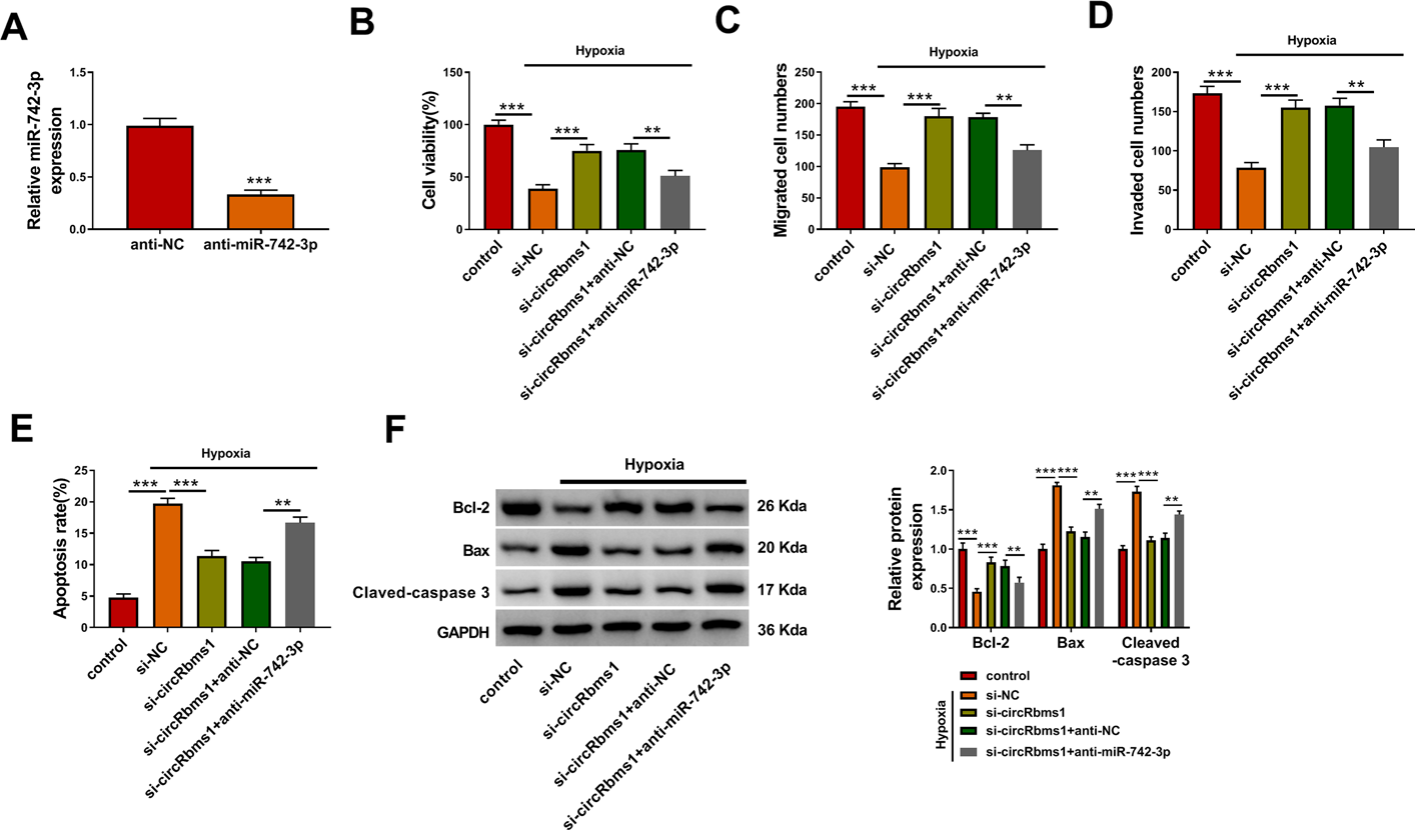
Effects of *circRbms1* silencing and *miR-742-3p* inhibitor on hypoxia-induced H9c2 cell injury. **A** After transfecting with anti-NC (50 nM) or anti-*miR-742-3p* (50 nM) into H9c2 cells, the expression of *miR-742-3p* was assessed by qRT-PCR. **B–F** H9c2 cells were transfected with si-NC (50 nM), si-*circRbms1* (50 nM), si-*circRbms1* (50 nM) + anti-NC (50 nM), or si-*circRbms1* (50 nM) + anti-*miR-742-3p* (50 nM), and then treated with hypoxia. Untreated H9c2 cells were used as control. CCK8 assay (**B**), transwell assay (**C**, **D**), and flow cytometry (**E**) were employed to examine cell viability, migrated and invaded cell numbers, and cell apoptosis rate, respectively. **F** WB analysis was used to test the protein levels of Bcl-2, Bax, and Cleaved-caspase 3. All experiments were repeated three times. **P < 0.01, ***P < 0.001

To determine whether *circRbms1* regulated hypoxia-induced cardiomyocyte injury by sponging* miR-742-3p*, rescue experiments were performed. After the H9c2 cells were transfected with anti-*miR-742-3p*, *miR-742-3p* expression was indeed reduced (Fig. 5A), confirming the transfection effectiveness of anti-*miR-742-3p*. Then, H9c2 cells transfected with si-*circRbms1* and anti-*miR-742-3p* were treated with hypoxia. As shown in Fig. 5B–D, the promoting effects of *circRbms1* silencing on the viability, the migrated cell numbers, and the invaded cell numbers of hypoxia-induced H9c2 cells were abolished by *miR-742-3p* inhibitor. Also, *miR-742-3p *inhibitor reversed the suppressive effect of *circRbms1* knockdown on the apoptosis rate of hypoxia-induced H9c2 cells (Fig. 5E). By measuring the protein levels of Bcl-2, Bax, and Cleaved-caspase 3, we confirmed that the upregulatory effect of *circRbms1* silencing on Bcl-2 expression and the downregulatory effect on Bax and Cleaved-caspase 3 expression could be reversed by *miR-742-3p* inhibitor (Fig. 5F).

### *FOXO1* was targeted by *miR-742-3p*

**Fig. 6:**
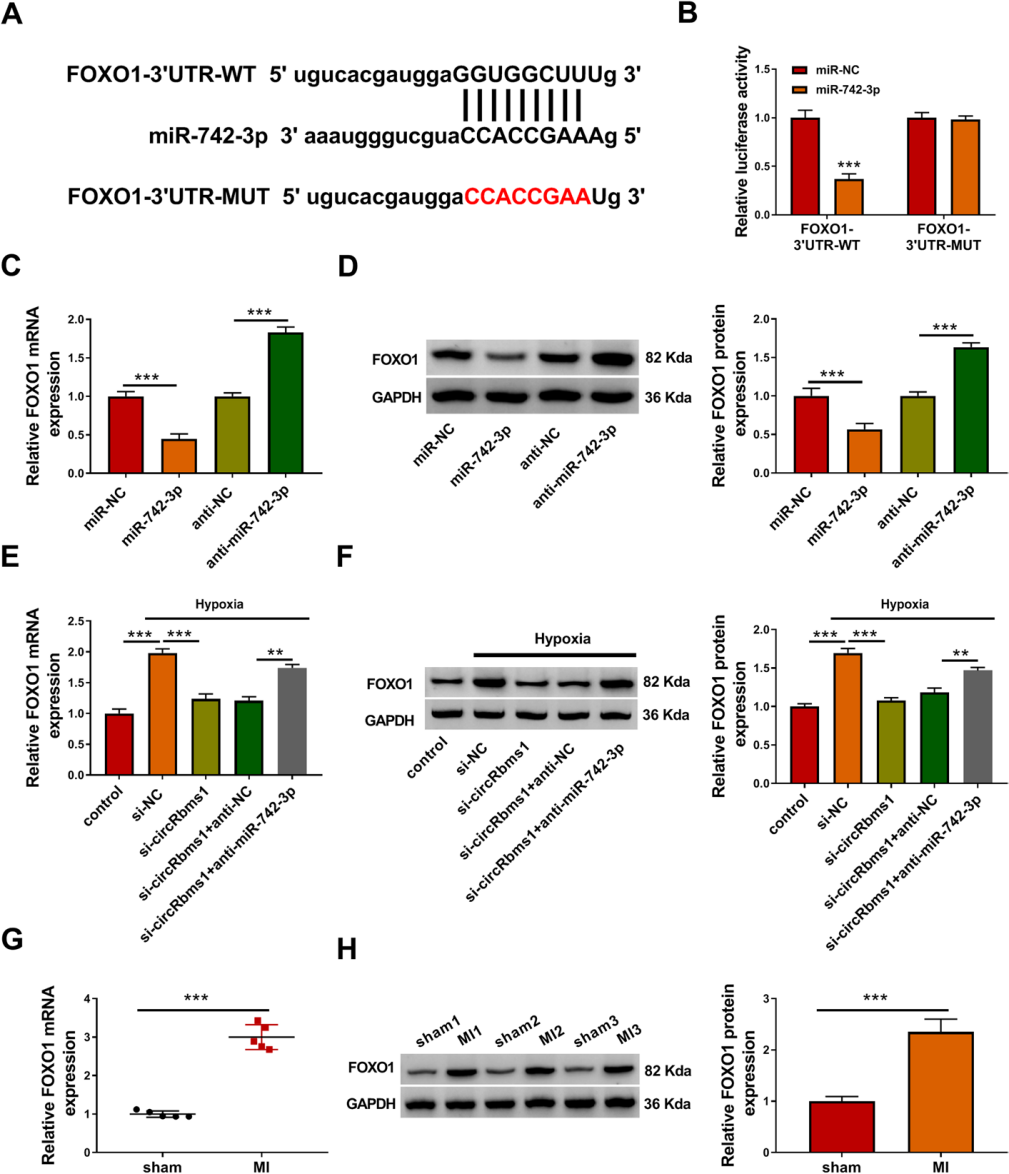
*FOXO1* was targeted by *miR-742-3p*. **A** The sequences of *FOXO1*-3′UTR-WT and *FOXO1*-3′UTR-MUT. **B** Dual-luciferase reporter assay was used to confirm the interaction between *miR-742-3p* and *FOXO1*. **C**, **D** H9c2 cells were transfected with miR-NC (50 nM), *miR-742-3p* (50 nM), anti-NC (50 nM), or anti-*miR-742-3p* (50 nM). The mRNA and protein expression levels of FOXO1 were measured using qRT-PCR and WB analysis. **E**, **F** H9c2 cells were transfected with si-NC (50 nM), si-*circRbms1* (50 nM), si-*circRbms1* (50 nM) + anti-NC (50 nM), or si-*circRbms1* (50 nM) + anti-*miR-742-3p* (50 nM), and then treated with hypoxia. Untreated H9c2 cells were used as control. qRT-PCR and WB analysis were used to determine the mRNA and protein expression levels of FOXO1. **G**–**H** The mRNA and protein expression levels of FOXO1 in the heart tissues of sham mice and MI mice were examined using qRT-PCR (*n* = 6 per group) and WB analysis (*n* = 3 per group). All experiments were repeated three times. **P < 0.01, ***P < 0.001

Furthermore, the Starbase tool also predicted that *miR-742-3p* could target the 3′UTR of *FOXO1* (Fig. 6A). Dual-luciferase reporter assay results revealed that the *miR-742-3p* overexpression could reduce the luciferase activity of *FOXO1*-3′UTR-WT vector, while not affecting that of the *FOXO1*-3′UTR-MUT vector (Fig. 6B). Through assessing the mRNA and protein expression levels of FOXO1, we discovered that FOXO1 expression was markedly inhibited by *miR-742-3p* overexpression and notably enhanced by* miR-742-3p* inhibition (Fig. 6C, D). Meanwhile, *circRbms1* knockdown inhibited the mRNA and protein expression of FOXO1 promoted by hypoxia, while this effect was also reversed by *miR-742-3p* inhibitor (Fig. 6E, F). Additionally, we measured FOXO1 expression in the heart tissues of MI mice, and found that it was remarkably upregulated compared with the sham group (Fig. 6G, H).

### Overexpressed FOXO1 partially reversed the regulation of *miR-742-3p* on hypoxia-induced H9c2 cell injury

**Fig. 7:**
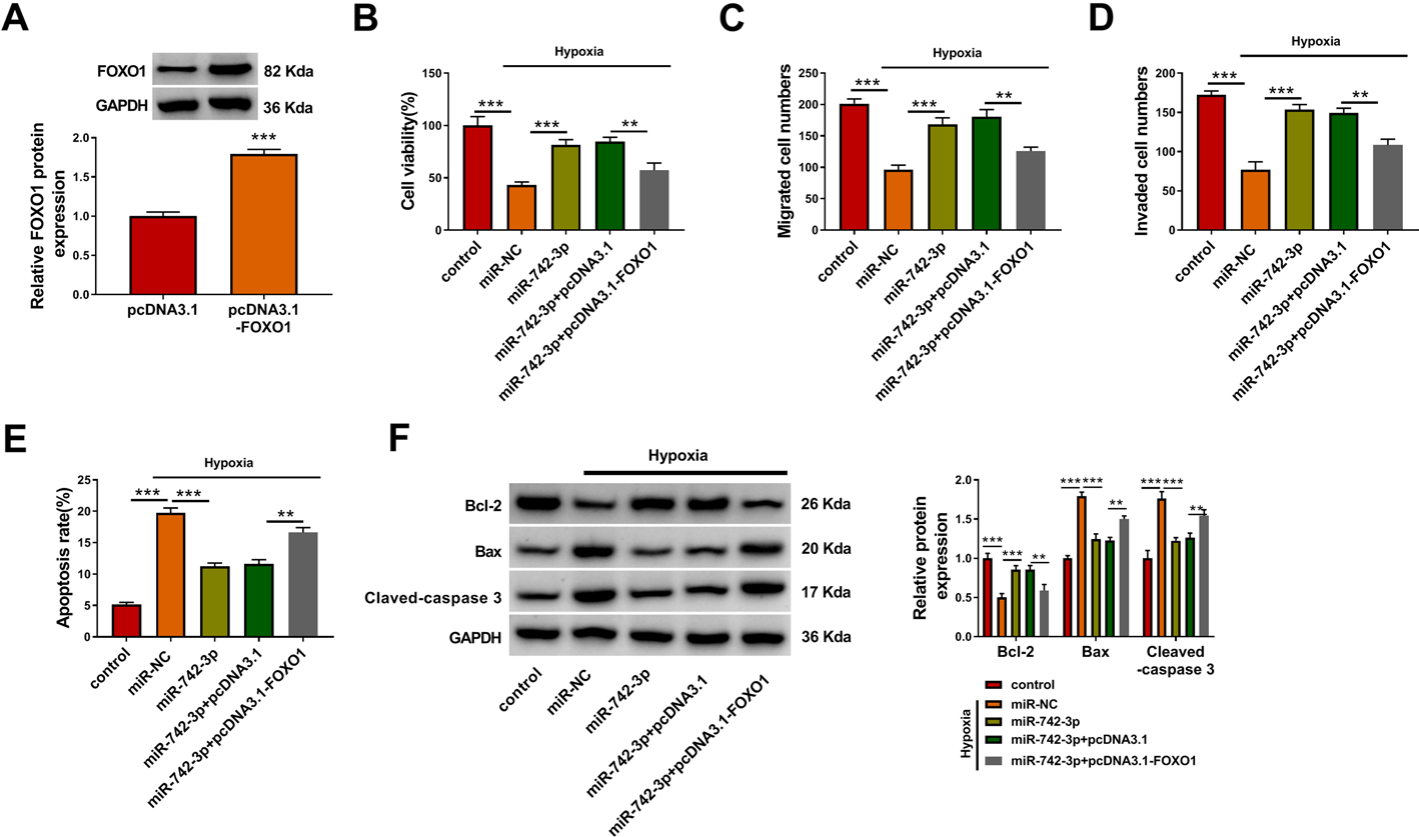
Effects of *miR-742-3p* and *FOXO1* on hypoxia-induced H9c2 cell injury. **A** H9c2 cells were transfected with pcDNA3.1 and pcDNA3.1-*FOXO1*, and the protein expression of FOXO1 was detected by WB analysis. **B**–**F** H9c2 cells were transfected with miR-NC (50 nM), *miR-742-3p* (50 nM), *miR-742-3p *(50 nM) + pcDNA3.1 (4.0 µg), or *miR-742-3p* (50 nM) + pcDNA3.1-*FOXO1* (4.0 µg), and then treated with hypoxia. Untreated H9c2 cells were used as control. Cell viability, migrated and invaded cell numbers, and cell apoptosis rate were determined by CCK8 assay (**B**), transwell assay (**C**, **D**), and flow cytometry (**E**). **F** WB analysis was employed to examine the protein levels of Bcl-2, Bax and Cleaved-caspase 3. All experiments were repeated three times. **P < 0.01, ***P < 0.001

**Fig. 8:**
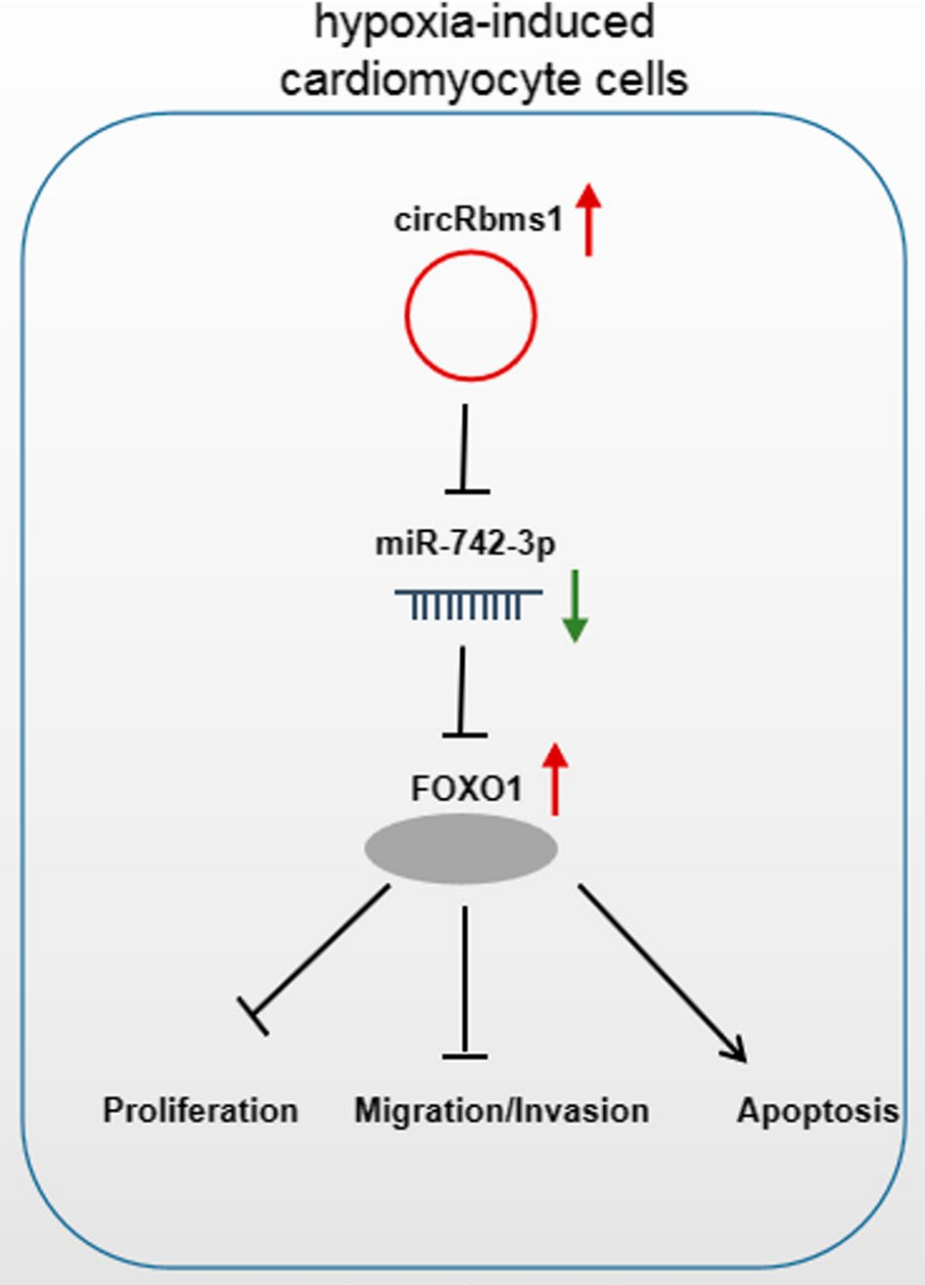
Mechanism diagram for this study. In hypoxia-induced cardiomyocyte cells, *circRbms1* inhibited proliferation, migration, and invasion, while promoting apoptosis by regulating *miR-742-3p*/*FOXO1*

To confirm that *miR-742-3p* regulated hypoxia-induced H9c2 cell injury via targeting *FOXO1*, we constructed the pcDNA3.1 *FOXO1 *overexpression vector to carry out the rescue experiments. The increased FOXO1 expression confirmed that the transfection efficiency of pcDNA3.1-*FOXO1* was good (Fig. 7A). In hypoxia-induced H9c2 cells co-transfected with *miR-742-3p* mimic and pcDNA3.1-*FOXO1*, we discovered that the enhancing effect of *miR-742-3p* on the viability, the migrated cell numbers, and the invaded cell numbers was abolished by FOXO1 overexpression (Fig. 7B–D). In addition, the inhibitory effect of *miR-742-3p* on the apoptosis rate, the Bax and Cleaved-caspase 3 protein levels, and the promoting effect on Bcl-2 protein level was also reversed by FOXO1 overexpression (Fig. 7E, F). All results indicate that *miR-742-3p* alleviated hypoxia-induced H9c2 cell injury by regulating FOXO1. Above all, our data show that *circRbms1* sponged *miR-742-3p* to upregulate FOXO1, thereby inhibiting proliferation, migration, and invasion and promoting apoptosis in hypoxia-induced cardiomyocyte cells (Fig. 8).

## Discussion

Cardiovascular disease is one of the major diseases that seriously affect public health. Therefore, exploring the pathogenesis of MI is of great significance for disease diagnosis and treatment. In this study, we explored the role of a newly discovered circRNA,* circRbms1*, which was screened in the GEO database to be differentially expressed in the heart tissues of MI mice. In MI mice and hypoxia-induced H9c2 cells, we confirmed that *circRbms1* had a significant high expression. Further experiments showed that overexpressed* circRbms1* aggravated hypoxia-induced cell injury in simulated cardiomyocyte, while its knockdown protected cardiomyocytes from hypoxia-induced injury. These findings confirm the key function of *circRbms1* in regulating cardiomyocyte injury and indicate that *circRbms1* might have clinical significance in the treatment of MI.

The ceRNA mechanism of circRNA has been confirmed in many studies [19, 20]. For example, *circ_28313* could act as a ceRNA for *miR-195a* to regulate osteoclast differentiation [21]. Zhang et al. reported that *circNRIP1* could facilitate gastric cancer proliferation and metastasis by sponging *miR-149-5p* [22]. *Circ_010567* was found to increase myocardial fibrosis through targeting *miR-141* [23]. In MI, *circCDYL* had been discovered to serve as a *miR-4793-5p* sponge to enhance cardiomyocyte proliferation [24]. Using bioinformatics analysis and experimental verification, we confirmed that *circRbms1* contained* miR-742-3p* binding sites. Past studies had shown that *miR-742-3p* was significantly underexpressed in the liver of obese mice, and might be associated with the progression of nonalcoholic fatty liver disease [25]. In our research, *miR-742-3p* was discovered to be lowly expressed in the heart tissues of MI mice and hypoxia-induced H9c2 cells. Gain-of-function experiments showed that *miR-742-3p* overexpression could relieve hypoxia-induced cardiomyocyte injury, suggesting that *miR-742-3p* might inhibit MI progression. Furthermore, *miR-742-3p* inhibitor reversed the effect of *circRbms1* knockdown on hypoxia-induced cardiomyocyte injury, indicating that *circRbms1* might participate in the regulation of MI progression via sponging *miR-742-3p*.

In addition, *FOXO1* was confirmed to be a target of *miR-742-3p*. FOXO1 is a member of the O subgroup of the FOX family and is involved in regulating various biological processes, including oxidative stress, proliferation, and apoptosis [26, 27]. In addition, FOXO1 has been shown to play a vital role in embryonic development, fat formation, and tumor formation [28–30]. Ma et al. showed that FOXO1 could increase hypoxia–reoxygenation cardiomyocyte injury [31], and Qiu et al. proposed that knockdown of FOXO1 could inhibit hydrogen-peroxide-induced cardiomyocyte oxidative stress and apoptosis [32]. Here, we confirmed that FOXO1 was upregulated in the heart tissues of MI mice and hypoxia-induced H9c2 cells, and found that *circRbms1* sponged *miR-742-3p* to positively regulate FOXO1. Further analysis verified that *miR-742-3p* targeted FOXO1 to relieve hypoxia-induced cardiomyocyte injury. The pro-cardiomyocyte injury effect of FOXO1 was also demonstrated in our study.

Of course, our study has some limitations. Although *mmu_circ_0001022* is the homologous circRNA of *hsa_circ_0056866* in mice, we have not verified the function and mechanism of *hsa_circ_0056866 *in human cardiomyocytes. Therefore, more research is needed to explore the function of *circRbms1* in human cardiomyocytes to further determine its feasibility as a therapeutic target for MI.

In summary, our study revealed the role of a new circRNA in MI progression. This research suggests that silenced *circRbms1* could alleviate cardiomyocyte injury after hypoxia by regulating the *miR-742-3p*/*FOXO1* axis. Our findings provide a potential target for MI treatment and a reference for the study of *circRbms1*.

## Supplementary Information

**Additional file 1: Fig. S1. **Effects of *circRbms1* knockdown on cell cycle process in hypoxia-induced H9c2 cells. H9c2 cells were transfected with or without si-NC (50 nM), si-*circRbms1* (50 nM), vector (4.0 µg), or *circRbms1* (4.0 µg), and then treated with hypoxia. Untreated H9c2 cells were used as control. **A**, **B** Flow cytometry was used to assess the cell cycle distribution. All experiments were repeated three times. ***P < 0.001

## Data Availability

Please contact the corresponding author for data requests.

## Abbreviations

circRNA: circular RNA
MI: myocardial infarction
miR: microRNA
FOXO1: forkhead box O1
qRT-PCR: quantitative real-time PCR
CCK8: Cell Counting Kit-8
WB: western blot
ActD: actinomycin D
WT: wild type
MUT: mutant type
