# Supplementary figures and images for: CircRbms1 knockdown alleviates hypoxia-induced cardiomyocyte injury via regulating the miR-742-3p/FOXO1 axis
Source: Cell Mol Biol Lett. 2022 Mar 26;27:31. doi: 10.1186/s11658-022-00330-y (PMC8962532; doi:10.1186/s11658-022-00330-y)

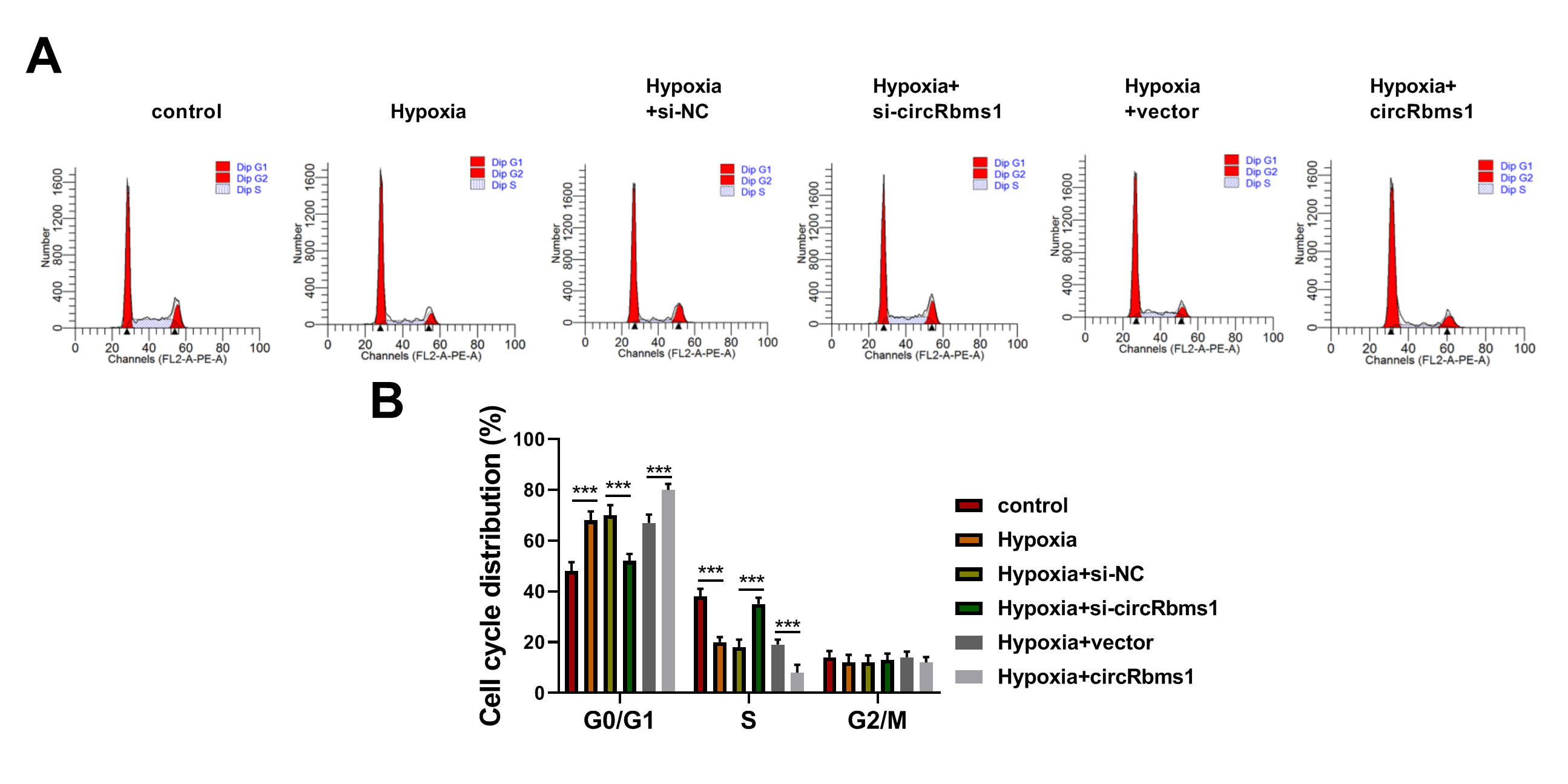

Supplement: Supplementary file 1 — Additional file 1: Fig. S1. Effects of circRbms1 knockdown on cell cycle process in hypoxia-induced H9c2 cells. H9c2 cells were transfected with or without si-NC (50 nM), si-circRbms1 (50 nM), vector (4.0 µg), or circRbms1 (4.0 µg), and then treated with hypoxia. Untreated H9c2 cells were used as control. A, B Flow cytometry was used to assess the cell cycle distribution. All experiments were repeated three times. ***P < 0.001 [file 11658_2022_330_MOESM1_ESM.tif]
